# *Ocimum basilicum* Essential Oil Modulates Hematotoxicity, Oxidative Stress, DNA Damage, and Cell Cycle Arrest Induced by β-cyfluthrin in Rat Liver

**DOI:** 10.3389/fphar.2021.784281

**Published:** 2022-01-21

**Authors:** Ali B. Jebur, Raghda A. El-Sayed, Fatma M. El-Demerdash

**Affiliations:** ^1^ Department of Animal Production, College of Agriculture, University of Kerbala, Kerbala, Iraq; ^2^ Department of Environmental Studies, Institute of Graduate Studies and Research, Alexandria University, Alexandria, Egypt

**Keywords:** *Ocimum basilicum* leaves extract, β-cyfluthrin, oxidative stress, enzymes, rat liver, molecular changes

## Abstract

Pesticides are used in large quantities infrequently, resulting in environmental damage and health issues. The goal of the current study was to explore the ameliorating effect of *Ocimum basilicum* (Basil) leaves essential oil versus the harmful effects of β-cyfluthrin in rat liver. Male Wistar rats were classified at random into four groups; negative control (corn oil), basil leaves essential oil (BEO, 3 ml/kg), β-cyfluthrin (positive control) (β-Cyf; 15 mg/kg BW, 1/25 LD_50_), and BEO plus β-Cyf, respectively. The rats were given their doses orally every day for a month. Results revealed that BEO yielded 6.32 mg/g with 33 identified components, representing 97% of the total oil. BEO implicated a considerable level of total phenolic contents, DPPH radical scavenging capacity, ABTS activity, and FRAP. The treatment of β-Cyf dramatically elevated lipid peroxidation (TBARS and H_2_O_2_) (LPO), protein oxidation (PC, AOPP, and HYP), and considerably reduced enzymatic (SOD, CAT, GPx, GR, and GST) and non-enzymatic (GSH) antioxidants. After β-Cyf treatment, hematological parameters, body and liver weights, enzyme activity (AST, ALT, ALP, and LDH), as well as protein, albumin, globulin, and total bilirubin levels were all considerably affected. Furthermore, β-Cyf increased the expression of pro-inflammatory genes (TNF-α, IL-6) as well as DNA damage and cell cycle arrest in the G0/G1 phase and decreased the number of cells in S and G2/M phase of liver cells. Moreover, rats given BEO then intoxicated with β-Cyf showed substantial changes in the majority of the parameters tested. Finally, BEO was shown to have high antioxidant efficacy in combating β-Cyf toxicity because of its high phenolic content.

## Introduction

Pyrethroids insecticides, synthetic derivatives of pyrethrins, are utilized worldwide because of their high toxicity to insects and minimal efficiency in mammals (Das et al., 2017; Tang et al., 2018). β-cyfluthrin (β-Cyf), a photostable type II pyrethroid insecticide, is widely utilized in agriculture and household applications (Wang et al., 2020). Workers may be exhibited to β-Cyf as a consequence of occupational exposure; pest control operations and contaminated food and water (Gorell et al., 1998). The mechanism of action was discovered to be the interaction of β-Cyf with sodium ion-gated channels, which leads to membrane depolarization and loss of electrical excitability in the nervous system. Another mechanism is linked to the disruption in calcium concentration in neurons through the inhibition of calcium transporter enzymes (Wolansky et al., 2006). Furthermore, different data revealed that pyrethroids caused oxidative stress and generated reactive oxygen species (ROS) during their metabolism in mammals (Jebur et al., 2014), leading to increased lipid peroxidation (Afolabi et al., 2019).

**GRAPHICAL ABSTRACT:**
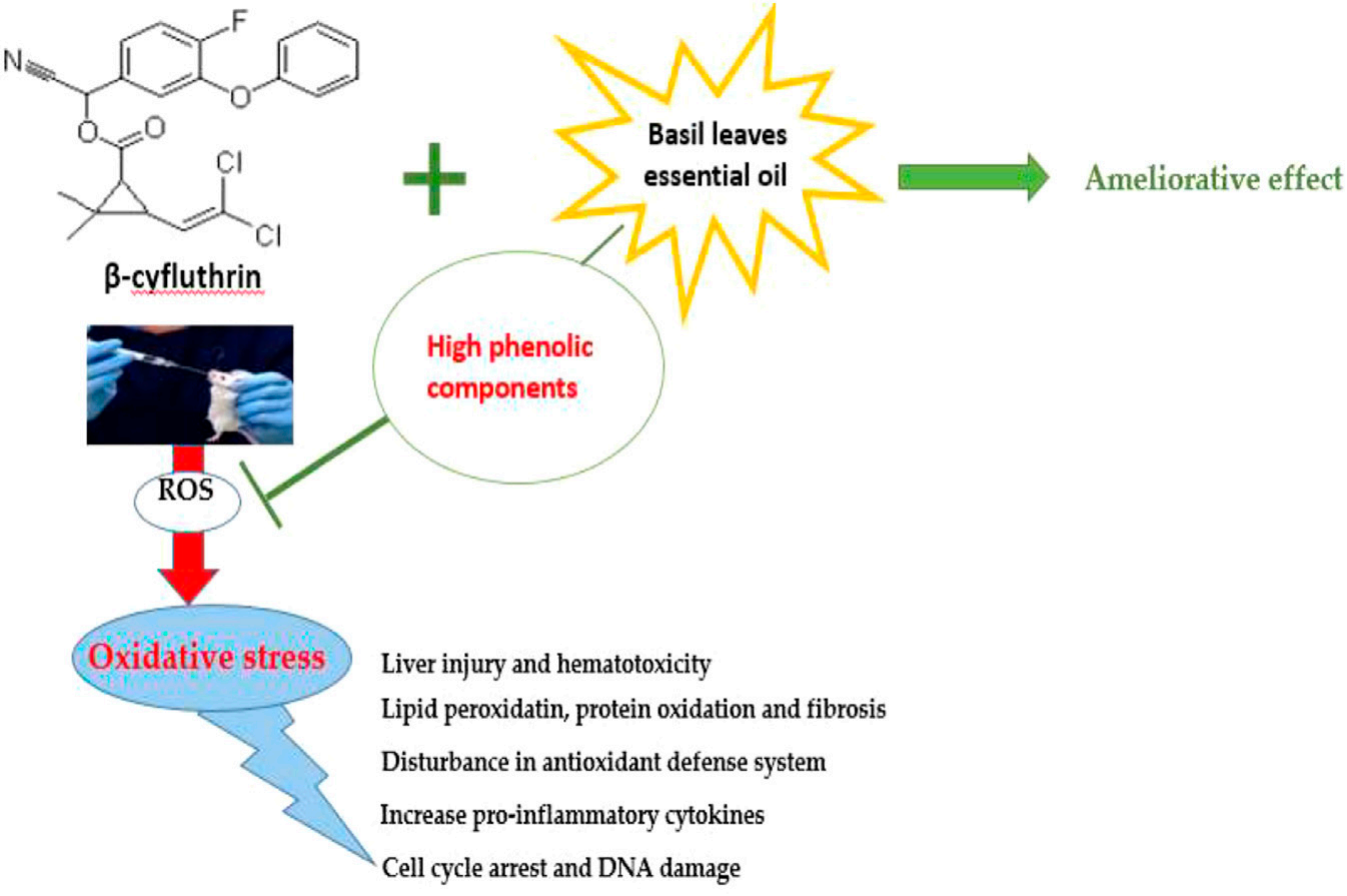
β–Cyf and BEO possible mechanisms.

The botanical medicinal products with abundant phenolic components are gaining popularity because of their potential to sweep free radicals (Gülçin et al., 2007). Basil; the common name of *Ocimum basilicum* L.; is a herbaceous and aromatic plant from the Lamiaceae Martinov family, Genus: Ocimum L. and is cultivated all over the world (Shirazi et al., 2014). Basil is regarded as one of the most effective botanical medications for treating a variety of ailments (Patel et al., 2018).

Antioxidant, anti-aging, anti-cancer, antiviral, antibacterial, anti-genotoxic, and anti-inflammatory properties are among its many benefits (Teofilović et al., 2021). Also, basil has been demonstrated to have a variety of pharmacological effects in many disorders, including hepatic fibrosis (Alomar and Al-Attar, 2019), diabetes (Widjaja and Rusdiana, 2019), asthma (Eftekhar et al., 2019), anemia (Zangeneh et al., 2019), and brain damage (Singh et al., 2018). Furthermore, simultaneous therapy with *O. basilicum* protects against oxidative stress caused by Mercury chloride and cadmium (Sakr and Nooh, 2013; Sunday et al., 2018), as well as deltamethrin. (Sakr and Al-Amoudi, 2012). Therefore, the goal of this investigation was to assess the protective effects of *O. basilicum* essential oil against hepatotoxicity induced by β-cyfluthrin in rats.

## Materials and Methods

### Chemicals and Reagents

β-cyfluthrin (Purity >99%) was acquired from Nanjing Panfeng Chem Ltd. (Nanjing, Jiangsu, China). The rest of the reagents were analytical reagent grade. Fresh basil leaves (*Ocimum basilicum* L. (Lamiaceae) were obtained from the Faculty of Science, where they were recognized and authenticated as Basil; the common name of *Ocimum basilicum* L.; Lamiaceae Martinov family, Genus: Ocimum L. by a plant taxonomy specialist at the Department of Botany, Faculty of Science, Alexandria University, Alexandria, Egypt.

### Extraction of *O. basilicum* Leaves Essential Oil

Basil dry leaves (100 g) were crushed and hydrodistilated with sterile water (1 L) for 3 h using a Clevenger-type device. The essential oil was extracted and dried over anhydrous sodium sulfate before being kept at 4°C for further use.

### GC/MS Analysis of *O. basilicum* Leaves Essential Oils

A Thermo Scientific GC/MS version (5) 2009 system with TG-5MS column (30mX0.32mmID) was used to identify the chemical elements of the extracted essential oil. At a flow rate of 1 ml/min, helium was used as the carrier gas. Five μl essential oil was diluted to 1 ml with dichloromethane, then 2 μl was injected on splitless mode for 1 min, followed by a 1:10 split flow. GC oven temperature was held at 45°C for 2 min then was programmed from 45°C to 165°C at 4°C/min; from 165°C to 280°C at 15°C/min after which was kept constant at 280°C for 10 min. At 250°C, both the interface and injection temperatures were set. The ionization voltage was 70 eV with a mass range between 40–800 m/z (Adams, 2006). The components of the essential oil were identified using mass fragmentation patterns, which were compared to the mass spectral database (version 2) of the National Institute of Standards and Technology (NIST), and their relative percentages were estimated using GC peak regions.

### Determination of Total Phenolic Content

The total phenolic contents in BEO were determined spectrophotometrically using Folin-Ciocalteau method (Kang et al., 2010). The absorbance was measured at 765 nm against blank. The calibration curve was produced using pyrocatechol as a standard. The total phenolic content of the extract was measured in milligrams of pyrocatechol equivalents (PE) per Gram of extract.

### Determination of DPPH Free Radical Scavenging Activity

The reducing ability of antioxidants toward the DPPH (2,2-diphenyl-1-picryl-hydrazyl-hydrate) radical was estimated to determine the free radical scavenging activity of the sample extract (Kang et al., 2011). Using butylated hydroxytoluene (BHT) as a positive control, the DPPH radical scavenging activity of each sample was measured at 515 nm and computed as follows: percent inhibition = [(AB-AA)/AB] x 100, where AB and AA are the absorbance values of the control and test samples, respectively. The IC_50_ value is used to represent the radical-scavenging activity of DPPH.

### Determination of ABTS Radical Scavenging Assay

With slight adjustments, the antioxidant capacity was calculated using the approach reported by Re et al. (1999). A spectrophotometer was used to measure the absorbance at 734 nm. The percent inhibition of ABTS•+ was calculated for each test sample using the formula: (percent inhibition) = [100*(Ac–As/Ac)], where Ac represents the control sample absorbance and As represents the test sample absorbance. The amount of oil required to scavenge 50% of the ABTS radicals (IC_50_) was estimated. Trolox was utilized as a control substance.

### Determination of Ferric Reducing Antioxidant Power (FRAP)

As a measure of “antioxidant power,” a ferric reducing ability (FRAP) assay (Benzie and Strain, 1996) was used to estimate the total antioxidant capacity of the sample. The change in absorbance at 593 nm is measured in this experiment.

### Animals and Treatments

Twenty-eight male Wistar rats (150 ± 10 g) were given by the Faculty of Medicine, Alexandria University, Alexandria, Egypt. The local Research Ethical Committee accepted the experimental design, which followed the National Institutes of Health’s guidelines (NIH). The rats were kept in groups and fed rat chow and water *ad libitum* while being kept at 21–24°C and 40–60% relative humidity with 12-h light-dark cycles. Animals were randomly divided into four groups (*n* = 7/group) after 2 weeks of acclimatization. The first group was given corn oil (negative control), the second group received basil essential oil (BEO; 3 ml/kg), the third group (positive control) was treated with β-cyfluthrin (β-Cyf; 15 mg/kg/day; 1/25 LD_50_), and the fourth group-administered BEO an hour before β-Cyf intoxication. Dosages of β-Cyf (Singh et al., 2009) and BEO (Farghali et al., 2014) were given orally for 1 month daily. After the end of the treatment period, rats were anesthetized using isoflurane, euthanized and blood and livers were collected for further analysis. The liver was cut into three pieces: the first was held at 20°C (for biochemical assays), the second was kept at 80°C (for RNA extraction), and the third was used right away (for comet assay and flow cytometry).

### Body and Organ Weights

The body weights were recorded at the start of the treatment period (starting body weight) and at the end of the treatment period (final body weight). The livers were dissected and weighed after the associated tissues were cut away. In addition, organ relative weight was reported as g/100 g of body weight.

### Blood Samples

Blood samples were drawn through cardiac puncture and allowed to clot for 30 min at 25°C before being centrifuged for 15 min at 3000 g. Serum was taken and kept at −80°C until it was utilized to determine liver function indicators. Other blood samples were placed in EDTA tubes and analyzed using an automated analyzer for the identification of the complete blood count (CBC) (Sysmex kx-21n Automated Hematology Analyzer; JAPAN CARE CO., LTD.).

### Tissue Preparation

After rats’ dissection, the liver of each rat was taken and homogenized in sodium-potassium phosphate buffer (0.01 mol/L, pH 7.4). The homogenates were centrifuged for 20 min at 10,000 g (4°C), and the supernatants were used in various assays.

### Estimation of Lipid Peroxidation and Protein Oxidation Markers

Thiobarbituric acid-reactive substances (TBARS) and hydrogen peroxide (H_2_O_2_) were measured in liver homogenate according to the methods of Ohkawa et al. (1979) and Velikova et al. (2000), respectively. The concentrations of lipid hydroperoxides (LOOH) in liver homogenates were assessed using Nourooz-Zadeh et al. (1994) method. The oxidative protein damage was measured spectrophotometrically in the tissue by evaluating the advanced oxidized protein products (AOPP) (Witko-Sarsat et al., 1996) and protein carbonyl (PC) (Reznick and Packer, 1994). The levels of hydroxyproline (HYP) were assessed using the method of Bergman and Loxley (1963).

### Determination of Enzymatic and Non-Enzymatic Biomarkers

The content of reduced glutathione (GSH) in liver homogenates was determined using the method of Ellman (1959) after the reaction with 5,5′- dithiobis-(2-nitrobenzoic acid). The activities of glutathione peroxidase (GPx; EC 1.11.1.9) and glutathione reductase (GR; EC 1.6.4.2) were evaluated according to Hafeman et al. (1974) while superoxide dismutase (SOD; EC 1.15.1.1), catalase (CAT; EC 1.11.1.6), and glutathione S-transferase (GST; EC 2.5.1.18) were carried out using the methods of Misra and Fridovich (1972), Aebi (1984), and Habig et al. (1974), respectively.

### Determination of Liver Function Biomarkers

The activities of liver aminotransferases (AST; EC 2.6.1.1 and ALT; EC 2.6.1.2), alkaline phosphatase (ALP; EC 3.1.3.1), and lactate dehydrogenase (LDH; EC 1.1.1.27) activities, as well as total protein, albumin, and total bilirubin, were measured using commercially available kits from Biodiagnostic Company, Egypt.

### Molecular Analysis by Real-Time PCR

Real-time PCR was used to assess the relative expression of inflammation-related genes (TNF-α, IL-6) in the liver (qPCR). Total RNA was obtained using a commercial kit (Gene JET RNA Purification Kit), as directed by the manufacturer (Thermo Scientific, #K0731, United States). After evaluation of RNA level by Nanodrop (Quawell, Q3000, United States), cDNA was produced by reverse transcription using a commercial kit (RevertAid H Minus Reverse Transcriptase) as described in the manufacturer’s instruction (Thermo Scientific, # EP0451, United States). The qPCR was performed using StepOnePlus real-time PCR system (Applied Biosystem, United States) and a mixture of cDNA, 2X Maxima SYBR Green Master Mix (Thermo Scientific, #K0221, United States), and gene-specific primers (Table 1). The β-actin gene was utilized as a reference (internal control) to calculate the fold change in target genes. The relative gene expression was calculated using the 2–ΔΔCt method The threshold cycle numbers (Ct) of the target gene were normalized to that of reference (ref.) gene, in both the test groups and the control group by using the following equations:

ΔCt test = Ct (target in test groups)—Ct (ref in test groups)
ΔCt calibrator = Ct (target in control)—Ct (ref in control)
The ΔCt of the test genes were normalized to the ΔCt of the calibrator:
ΔΔCt = ΔCt (Test)—ΔCt (Calibrator)
Fold change of relative gene expression was calculated as follow:
Fold Change = 2^–ΔΔCt^

**TABLE 1:** Forward and reverse primers sequence used in qPCR.

Gene	Forward primer	Reverse primer
**Inflammation**		
*IL-6*	CGG​CTA​CCA​CAT​CCA​AGG​AA	GCTGGAATTACCGCGGCT
*TNFα*	GCA​TGA​TCC​GCG​ACG​TGG​AA	AGA​TCC​ATG​CCG​TTG​GCC​AG
Housekeeping		
*β actin*	AAG​TCC​CTC​ACC​CTC​CCA​AAA​G	AAG​CAA​TGC​TGT​CAC​CTT​CCC

### Determination of DNA Damage by Comet Assay

Alkaline single-cell gel electrophoresis was used to measure DNA damage parameters in liver tissues including tail length, percentage of degraded DNA, and tail moment (comet assay). The DNA fragment movement patterns of 100 cells for each dose level were investigated using GelRed stain (red fluorescence) and a 40× objective on a fluorescent microscope with an excitation filter of 420–490 nm (issue 510 nm). Komet 5 image analysis software was used to accomplish the imaging (Kinetic Imaging, Liverpool, United Kingdom).

### Cell Cycle Analysis by Flow Cytometry

Flow cytometry was used to determine the influence of various treatments on the number of cells in each phase of the cell cycle, and it was carried out as previously described by Abdelwahab et al. (2019) with minor changes. Collagenase enzyme (0.1 percent, Invitrogen, United States) was used to lyse freshly dissected liver tissues, which were subsequently mesh filtered (0.7 mm nylon) and centrifuged (4,000 rpm/5 min). The cells were fixed and stained with propidium iodide before being examined with an Attune flow cytometer (Applied Bio-system, United States). Each cell cycle phase’s number of cells was counted and expressed as a percentage of the total number of cells.

### Statistical Analysis

Results from all groups were represented as means with standard errors (SEM) and analyzed with SPSS software (version 22, IBM Co., Armonk, NY). ANOVA and Tukey’s post-hoc test were used to compare between groups. The sample size (7 rats/group) used with Cohen’s d of two achieved power of 92.91% via Two- Sample *t*-test using Effect Size (PASS program version 15). *p*-values ≤ 0.05 were deemed significant.

## Results

### Essential Oil Yield and Chemical Composition

**FIGURE 1:**
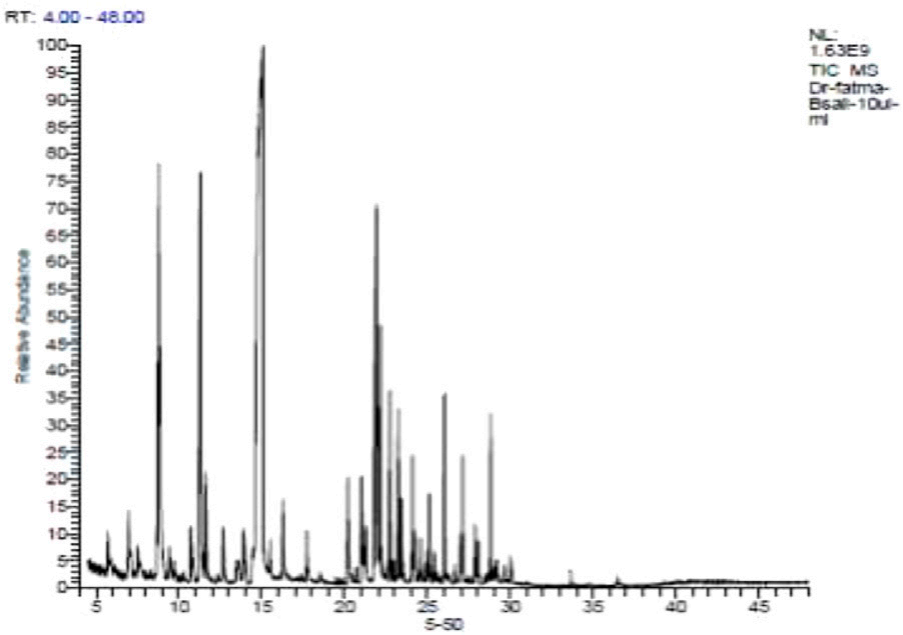
GC-MS chromatogram of basil essential oil.

The overall content of the basil essential oil (yield = 6.32 mg/g) contained 33 compounds, accounting for 97.0% of the total oil (Figure 1; Table 2), including monoterpenes, sesquiterpenes compounds, and phenylpropanoid derivatives. Estragole was the main component (28.6%). Linalool, the second most significant molecule, had a peak area of 21.7%. Other notable chemicals included (E)-methyl cinnamate (14.3%), α-cadinol (7.1%), eugenol (5.9%), 1,8-cineole (4.0%), methyl eugenol (3.1%), and α-bergamotene (2.2%).

**TABLE 2:** Chemical composition of *O. basilicum* essential oil.

No	Identified compound	RT	Peak area (%)	KI
1	β-Pinene	5.71	0.1	949
2	Limonene	7.52	0.1	1,005
3	1,8-Cineole	8.78	4.0	1,006
4	Camphor	9.41	0.5	1,109
5	Linalool	11.3	21.7	1,092
6	Bornyl acetate	11.62	0.5	1,252
7	Terpinen-4-ol	12.66	0.7	1,154
8	α-Bergamotene	13.55	2.2	1,407
9	Caryophyllene	13.90	0.3	1,385
10	Aloaromadendrene	14.42	0.1	1,450
11	Estragole	15.7	28.6	1,177
12	α-Terpineol	15.50	1.0	1,176
13	Germacrene D	16.27	0.3	1,444
14	α-Humulene	17.72	0.2	1,417
15	Carvone	20.20	0.4	1,207
16	β-Cubebene	21.03	0.5	1,059
17	β-Burbonene	21.20	0.011	1,354
18	β-Elemene	21.28	0.3	1,364
19	ɣ-Cadinene	21.94	0.2	1,426
20	Calamenene	22.16	0.2	1,483
21	α-Amorphene	22.74	1.0	1,479
22	β-Farnesene	23.24	0.2	1,452
23	∆-Cadinene	23.42	0.1	1,486
24	α-Bisabolene	24.10	0.1	1,506
25	(Z)-Methyl cinnamate	24.24	1.6	1,281
26	Methyl eugenol	24.59	3.1	1,378
27	(E)-Methyl cinnamate	25.00	14.3	1,364
28	Spatulenol	25.12	0.8	1,619
29	Eugenol	25.42	5.9	1,368
30	Carvacrol	26.03	0.04	1814
31	α-Cadinol	27.00	7.1	1,614
32	α-Caryophyllene	27.14	0.2	1,489
33	Phytone	27.89	0.7	1874
Total 97%				

RT: Retention time. KI**:** Retention index: Kovats retention index relative to n-alkanes on column.

### Antioxidant Properties

In the present study, *O. basilicum* leaves essential oil showed relatively high phenolic content with 40.9 mg PE/g. In comparison to the standard, BEO has a comparatively strong DPPH radical scavenging capacity, with an IC_50_ value of 10.79 mg/ml. BHT acid had a strong IC_50_ value of 6.80 mg/ml as a control. According to the findings, the BEO appears to have high antiradical activity, which could be ascribed to its high phenolic content, which traps the cation radical ABTS^•+^ by supplying H^+^. BEO had an IC_50_ (ABTS) of 0.69 ± 0.020 mg/ml, while Trolox had an IC_50_ (ABTS) of 3.35 ± 0.003. Furthermore, the FRAP assay is quick and easy to carry out, and the reaction is repeatable and linearly proportional to the antioxidant content present. It is calculated by comparing the total amount of antioxidants to the sample’s reducing capability. BEO has high reducing power (FRAP) equal (IC_50_ = 1.378 g/L), but less than the reducing power of butylated hydroxytoluene (BHT) (IC_50_ = 0.908 g/L) (Table 3).

**TABLE 3:** Phytochemical analysis of BEO.

Test	BEO	BHT	Trolox
TPCs (mg PE/g)	40.9 ± 2.3	**-**	**-**
DPPH (mg/ml)	IC_50_ = 10.79 ± 0.907	IC_50_ = 6.80 ± 0.518	**-**
ABTS (mg/ml)	IC_50_ = 0.69 ± 0.020	**-**	IC_50_ = 3.35 ± 0.003
FRAP (g/L)	IC_50_ = 1.378 ± 0.006	IC_50_ = 0.908 ± 0.003	**-**

Values are expressed as means ± SD (each test was triplicate).

### Body and Relative Organs Weights

During the research period, no mortality occurred while burrowing, salivation, tremors, writhing, and convulsions, are only observed as a few clinical indications of poisoning. In rats exposed to β-Cyf, there was a substantial decrease in body weights and an increase in relative liver weights when compared to control rats. In comparison to the β-Cyf exposed group, rats supplemented with BEO then treated with β-Cyf showed considerable alleviation in these parameters. BEO did not generate any substantial changes in rats when given alone (Table 4).

**TABLE 4:** Liver and body weights in different groups.

Parameters (g)	Control (negative control)	BEO	β-Cyf (positive control)	BEO + β-cyf
Initial body weight	157 ± 2.42	158 ± 2.11	164 ± 3.67	162 ± 2.87
Final body weight	215 ± 3.46^a^	218 ± 2.14^a^	188 ± 3.67^c^	199 ± 3.62^b^
Body weight gain	58.16 ± 3.62^a^	60.12 ± 5.49^a^	24 ± 2.36^c^	37.70 ± 2.65^b^
Absolute liver weight	4.97 ± 0.016^c^	5.10 ± 0.021^c^	7.11 ± 0.042^a^	6.15 ± 0.033^b^
Relative organ weight	2.31 ± 0.031^c^	2.33 ± 0.031^c^	3.78 ± 0.044^a^	3.09 ± 0.011^b^

Values are expressed as means ± SE; *n* = 7/group. Mean values within a raw not sharing common superscript letters were significantly different, *p* < 0.05. Groups are compared as follows: BEO, and β–Cyf are compared to the negative control group while BEO + β-Cyf group are compared to the positive control, β-Cyf group. Bodyweight gain (g) = final body weight–initial body weight.

### Hematological Parameters

Results of the hematological parameters in rats treated with β-Cyf showed a significant drop in hemoglobin (Hb) concentration, red blood cells (RBC), hematocrit (Ht), and thrombocyte values and an increase in white blood cells (WBC) when compared to the control group. Administration with basil alone showed minor changes in the hematological parameters compared to the control group while administration of BEO before β-Cyf intoxication showed significant restoration in these parameters near the normal level in comparison to the β-Cyf group (Table 5).

**TABLE 5:** Hematological analysis in different groups.

Parameters	Groups			
	Control (negative control)	BEO	β-Cyf (positive control)	BEO + β-cyf
RBC (×10^6^/L)	7.48 ± 0.11^a^	7.81 ± 0.791^a^	4.26 ± 0.49^c^	6.50 ± 0.08^b^
Hb (g/dl)	14.40 ± 1.13^a^	14.5 ± 0.902^a^	8.1 ± 1.71^c^	12.63 ± 0.18^b^
Ht (%)	43.20 ± 5.23^a^	42.82 ± 0.516^a^	21.00 ± 2.15^c^	37.83 ± 3.04^b^
WBC (×10^6^/L)	5.59 ± 1.08^c^	5.22 ± 0.523^c^	16.13 ± 6.07^a^	8.65 ± 0.29^b^
Thrombocytes (×10^6^/L)	818 ± 36.5^a^	828 ± 29.18^a^	323 ± 22.5^c^	784 ± 20.23^b^

Values are expressed as means ± SE; *n* = 7/group. Mean values within a raw not sharing common superscript letters were significantly different, *pP* < 0.05. Groups are compared as follows: BEO and β–Cyf are compared to the negative control group while BEO + β-Cyf group are compared to the positive control, β-Cyf group. Abbreviations: RBCs; red blood cells, Hb; hemoglobin, Ht; hematocrit, and WBC; white blood cell.

### Lipid Peroxidation and Protein Oxidation

**FIGURE 2:**
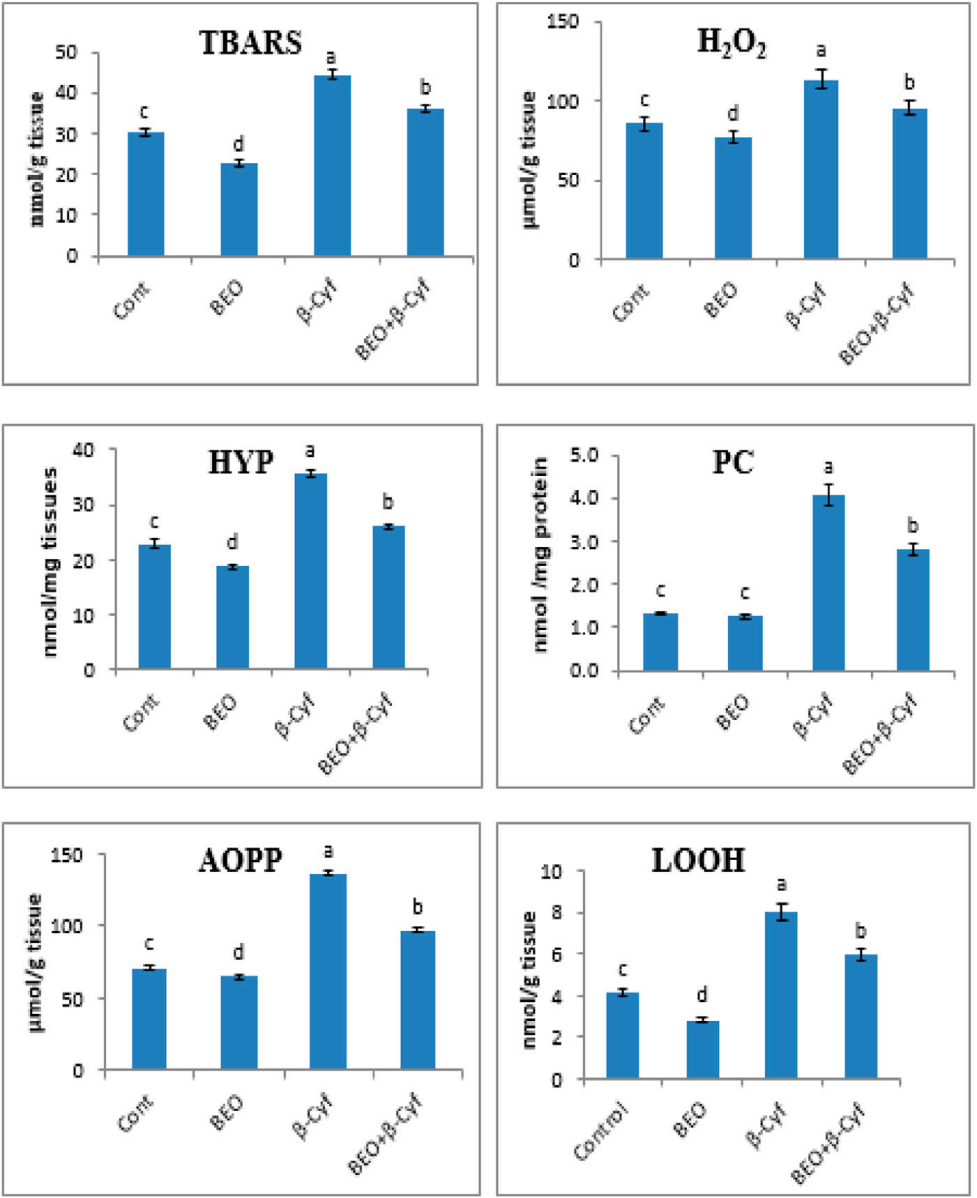
Lipid peroxidation and protein oxidation in the liver of male rats given BEO, β-Cyf, and BEO plus β–Cyf. Values are expressed as mean ± SE of seven rats per group. Columns with different letters are significantly different at *p* < 0.05. Groups are compared as follows: BEO and β–Cyf are compared to negative control while BEO + β-Cyf group are compared to the positive control group, β-Cyf. Abbreviations: TBARS, thiobarbituric acid reactive substances, H_2_O_2_; hydrogen peroxide, HYP; hepatic hydroxyproline, LOOH; lipid hydroperoxide, PC; protein carbonyl, AOPP; advanced oxidized protein product.

To examine the effects of β-Cyf administration on lipid peroxidation, TBARs, H_2_O_2,_ and lipid hydroperoxide (LOOH) levels were determined. Significant induction in TBARs, H_2_O_2,_ and LOOH concentrations after β-Cyf administration was detected as compared to the control group while rats who received BEO plus β-Cyf displayed a considerable decline in TBARS, H_2_O_2_, and LOOH concentrations as compared to the β-Cyf treated group. In addition, protein carbonyl (PC) and advanced oxidized protein products (AOPP) markers of protein oxidation were markedly increased in the liver of the β-Cyf intoxicated group. The increase in PC and AOPP levels in the liver was reduced by BEO administration. Hepatic hydroxyproline (HYP) levels were analyzed to evaluate hepatic collagen production after β-Cyf administration. The current data showed a significant increase in hepatic HYP levels in β-Cyf -treated rats in comparison to the control group. However, high levels of HYP levels dropped due to BEO administration before β-Cyf treatment. The liver of rats supplemented with BEO alone showed significant changes in the levels of most products of lipid peroxidation and protein oxidation as compared to the control group (Figure 2).

### Enzymatic and Non-Enzymatic Antioxidants

**FIGURE 3:**
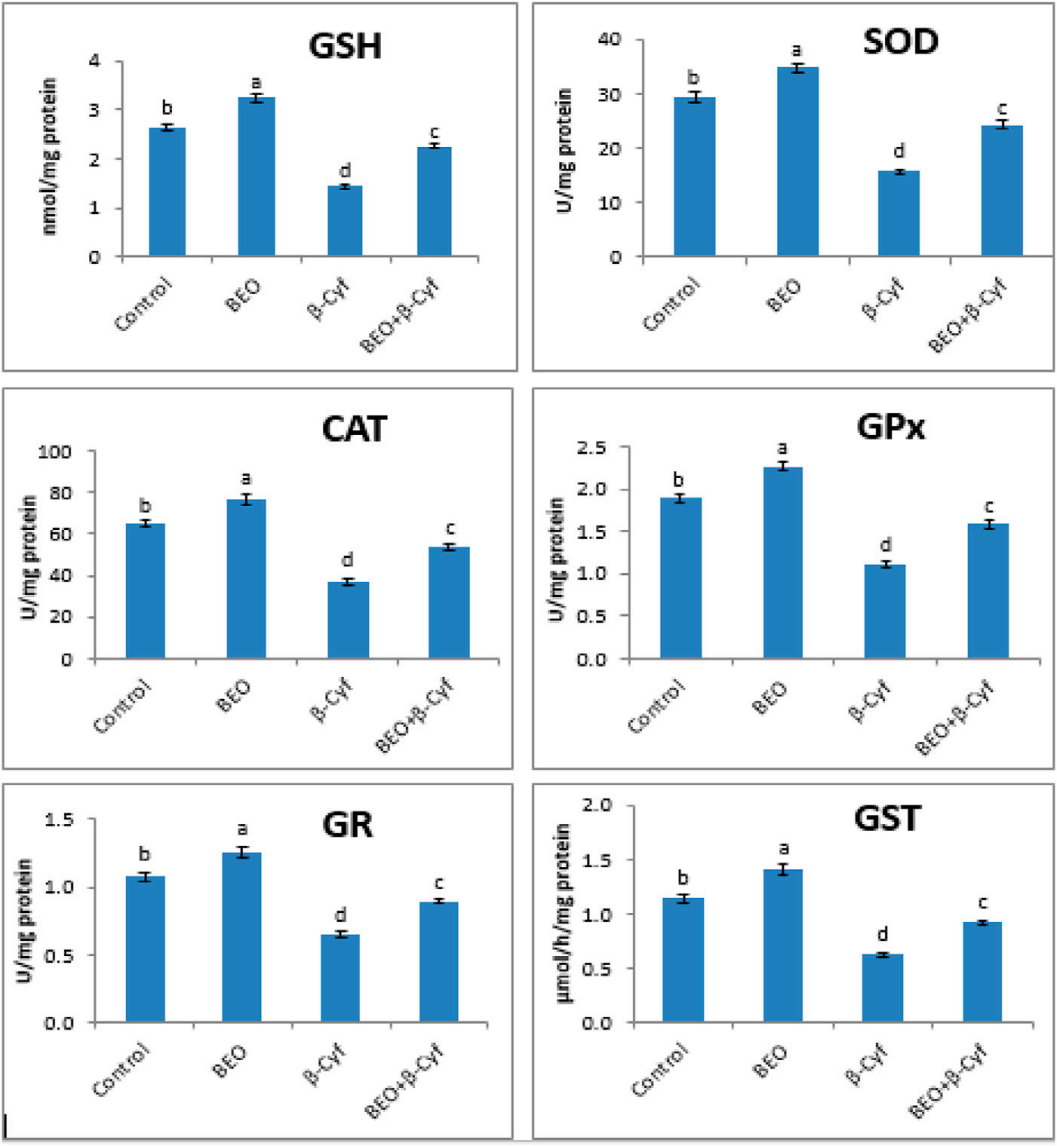
Reduced glutathione (GSH) content and activities of superoxide dismutase (SOD), catalase (CAT), glutathione peroxidase (GPx), glutathione reductase (GR), and glutathione S-transferase (GST) in the liver of male rats treated with BEO, β-Cyf, and BEO plus β–Cyf. Values are expressed as mean ± SE of seven rats per group. Columns with different letters are significantly different at *p* < 0.05. Groups are compared as follows: BEO and β–Cyf groups are compared to the negative control group while BEO + β-Cyf group is compared to the positive control group, β-Cyf.

Antioxidant enzyme activity (SOD, CAT, GPx, GR, and GST) and GSH levels are decreased significantly in liver homogenates of β-Cyf treated animals as compared to control. Otherwise, a significant induction in the same parameters was detected in the animals that received BEO plus β-Cyf as compared to the β-Cyf treated group. The use of BEO alone resulted in significant improvement in these indices (Figure 3).

### Liver Function Biomarkers

Hepatic enzymes as AST, ALT, ALP, and LDH are concerned as biomarkers of hepatotoxicity. Rats administered with β-Cyf exhibited alterations in AST, ALT, and ALP activities as well as protein content, total protein, albumin, globulin, and total bilirubin in both rat liver homogenate and serum as compared to the reference group (*p* < 0.05) while LDH activity is elevated significantly. Rats treated with BEO plus β-Cyf displayed significant modulation in enzyme activities and biochemical parameters as compared with the β-Cyf -treated group. Administration of BEO alone induced significant changes in some of these parameters (Table 6).

**TABLE 6:** Liver function biomarkers in rats in different groups.

Parameters	Groups			
	Control (negative control)	BEO	β-Cyf (positive control)	BEO + β-cyf
Liver				
AST (U/mg protein)	121 ± 3.65^a^	113 ± 3.91^b^	78 ± 2.76^d^	99 ± 3.24^c^
ALT (U/mg protein)	161 ± 5.92^a^	155 ± 4.31^ab^	102 ± 3.78^d^	137 ± 3.74^c^
ALP (U/mg protein)	388 ± 8.60^a^	365 ± 5.14^b^	261 ± 8.86^d^	335 ± 9.44^c^
LDH (U/mg protein)	793 ± 23.47^c^	823 ± 27.66^c^	1,063 ± 33.29^a^	891 ± 30.51^b^
Protein (mg/g tissue)	171 ± 3.68^b^	181 ± 6.44^a^	120 ± 4.09^d^	151 ± 2.72^c^
Serum				
AST (U/l)	53.40 ± 2.10^bc^	48.80 ± 4.21^c^	71.16 ± 2.28^a^	61.80 ± 2.36^b^
ALT (U/l)	54.80 ± 1.93^c^	46.0 ± 1.14^d^	76.80 ± 2.29^a^	65.0 ± 4.05^b^
ALP (U/l)	56.60 ± 0.96^c^	49.25 ± 1.11^d^	80.55 ± 0.80^a^	66.40 ± 2.37^b^
LDH (U/l)	625 ± 15.89^c^	584 ± 18.71^c^	869 ± 22.78^a^	692 ± 22.04^b^
Total protein (g/dl)	6.94 ± 0.24^b^	7.76 ± 0.18^a^	4.87 ± 0.13^d^	6.29 ± 0.11^bc^
Albumin (g/dl)	4.07 ± 0.14^ab^	4.48 ± 0.12^a^	2.94 ± 0.14^c^	3.80 ± 0.17^b^
Globulin (g/dl)	2.66 ± 0.10^ab^	2.90 ± 0.07^a^	2.22 ± 0.10^c^	2.50 ± 0.09^b^
Total bilirubin (mg/dl)	0.94 ± 0.05^c^	1.01 ± 0.06^c^	1.86 ± 0.11^a^	1.19 ± 0.10^b^

Values are expressed as means ± SE; *n* = 7/group. Mean values within a raw not sharing common superscript letters were significantly different, *p* < 0.05. Groups are compared as follows: BEO, and β–Cyf are compared to the negative control group while BEO + β-Cyf group are compared to the positive control, β-Cyf group.

### Pro-Inflammatory Cytokines

**FIGURE 4:**
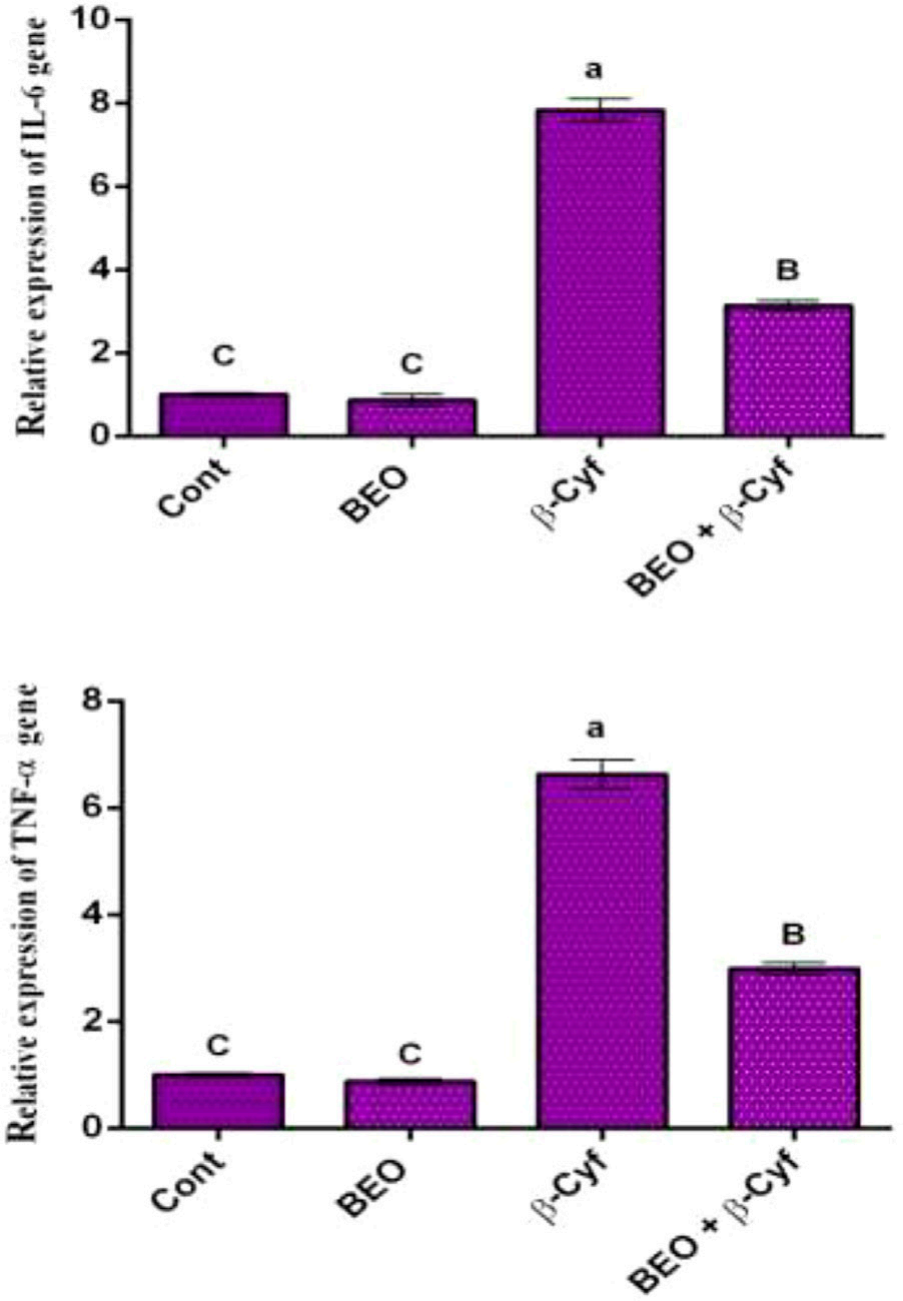
Relative expression of *IL-6* and TNF-α genes in rats’ liver tissues of different groups. Data are presented as fold change (mean) ± SEM (*n* = 7/group). Different letters show significant differences at *p* < 0.05. Groups are compared as follows: BEO and β–Cyf groups are compared to the negative control group while BEO + β-Cyf group is compared to the positive control group, β-Cyf.

In the liver tissue of rats treated with β-Cyf, qPCR results demonstrated significant increases in mRNA levels of pro-inflammatory cytokines TNF-α and IL-6 when compared to the control group. Animals pretreated with BEO then intoxicated with β-Cyf showed significant downregulation in the studied genes (Figure 4).

### DNA Damage

**FIGURE 5:**
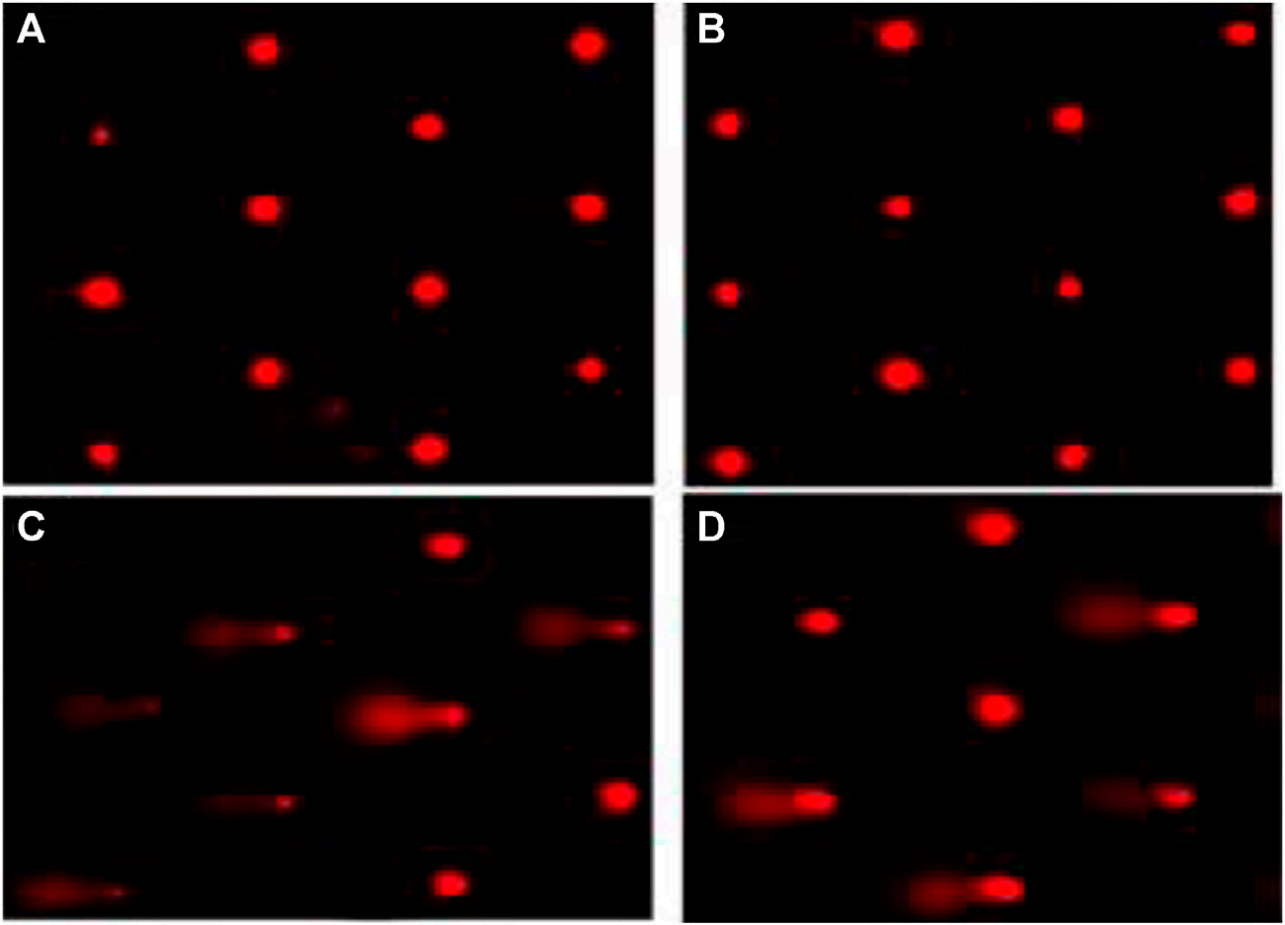
Liver DNA damage detected by the comet assay. **(A)**: cont. **(B)**: BEO; **(C)**: β-Cyf and **(D)**: BEO plus β–Cyf. Groups are compared as follows: BEO and β–Cyf groups are compared to the negative control group while BEO + β-Cyf group is compared to the positive control group, β-Cyf.

The DNA damage in the liver of rats in different groups was assessed using a comet test. In comparison to control (G1) and other treated groups, administration of β-Cyf (G3) resulted in a substantial increase in DNA damage (*p* < 0.05), as evidenced by an increase in tail length and tail moment. Because BEO was given before β-Cyf intoxication, the induction of DNA injury was reduced (G4). The control group (G1) and the BEO group (G2), on the other hand, showed no difference in DNA damage (tail length) (Figure 5).

### Cell Cycle Phase

**FIGURE 6:**
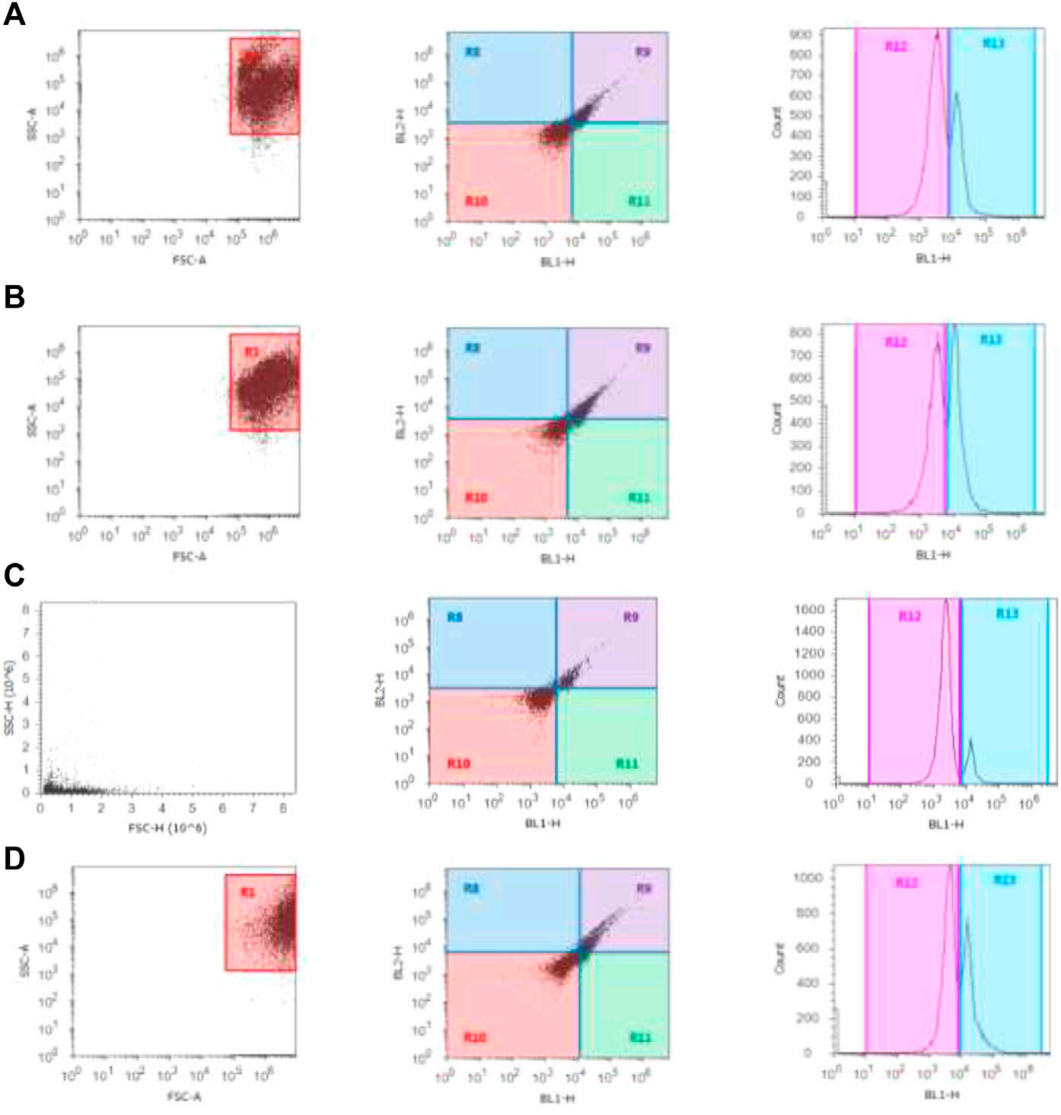
Cell cycle scatter and histograms for different groups **(A–D)**. The forward scatter (FS) and side scatter (SS) were measured to identify single cells. The *X*-axis in the histogram depicts propidium iodide fluorescence as a function of DNA content, while *Y*-axis reflects cells count in the 3 cell cycle phases (G0/G1, S, and G2/M).

The cells in the G0/G1 phase were arrested after treatment with β-Cyf, as evidenced by a considerable (*p* < 0.05) increase in the number of cells in this phase compared to the reference group. However, the β-Cyf treatment significantly reduced the number of cells in the S phase and G2/M phase (*p* < 0.05). Further, the number of cells in each cell cycle was recovered to a level comparable to the control group after treatment with BEO (Figure 6).

## Discussion

The use of pyrethroids in large quantities has increased the risks to the environment and human health. Several studies have shown that oxidative stress has a role in pyrethroid toxicity *in vivo* and *in vitro* (Prasanthi et al., 2005; Jebur et al., 2014). The toxic effect of β-Cyf on the gastrointestinal tract, which results in a decrease in food consumption and nutritional absorption, could explain the observed drop in rat body weight (Mohafrash et al., 2017). Furthermore, the enhanced cell division as an adaptive strategy of the liver to improve its function could be connected to the increase in relative liver weight in β-Cyf treated rats (Rifat et al., 2012). Also, the observed drop in RBCs count and Hb concentration could be attributable to excessive erythrocyte collapse and/or β-Cyf toxic effect on bone marrow (Verma et al., 2013). Furthermore, leukocytosis can be thought of as the body’s response to a strange material entering the body (Saxena and Tomar, 2003). So, the alteration in hematological parameters could be due to the direct harmful effect of β-Cyf.

Pyrethroids caused cellular oxidative damage and the buildup of peroxidation products, which are commonly utilized as oxidative stress markers (El-Demerdash, 2011; Mossa et al., 2013). In the present investigation, β-Cyf exposure caused oxidative stress, as demonstrated by significant induction in lipid peroxidation and protein oxidation products, such as TBARs, H_2_O_2_, PC, LOOH, and AOPP. Jebur et al. (2014) suggested that the increased levels of free radicals and weakened antioxidant defenses indicated that β-Cyf had the potential to cause cellular damage by reducing membrane mobility. Furthermore, because Type II pyrethroids are hydrophobic esters with a cyano group, β-Cyf toxicity is most likely caused by the release of unstable metabolites called cyanohydrins, which result in the creation of free radicals (El-Demerdash, 2007). Additionally, pyrethroid-induced oxidative toxicity is likely due to their lipophilicity, which allows them to easily percolate through cellular membranes (Nasuti et al., 2003). Liver fibrosis is characterized by the buildup of collagen in the liver, which is an unusual feature (Bissel et al., 1990). The observed increase in HYP; the main characteristic ingredient in collagen; reflects the amount of collagen and conveys the extent of fibrosis induced by β-Cyf (Hanauske-Abel, 1996).

Glutathione is the most important antioxidant in the antioxidant defense system, as it keeps cells’ redox balance in check and protects them from free radicals. GSH aids in ROS elimination through its action as a non-enzymatic antiradical and as a substrate for certain enzymes such as GPx (Tsukamoto et al., 2002). Many authors have indicated that the absence of sulfhydryl groups puts cells at oxidative stress risk (Bizzozero et al., 2006; Rosa et al., 2007). Higher ROS production, as demonstrated by increased LPO, could explain the detected drop in GSH level as well as GPx and GR activities in β-Cyf treated rats. Enzymatic antioxidants such as SOD and CAT are also thought to be the first line of defense against the damaging effects of ROS generated by pyrethroids on biological macromolecules (El-Demerdash, 2007). Due to its change by xenobiotics, GST, a phase II enzyme, acts as a biomarker; also, it binds and removes metabolites produced by phase I enzymes of the cytochrome P_450_ monooxygenase system. Further, the observed decrease in GST activity caused by β-Cyf could be due to GSH depletion via a detoxifying mechanism (Ranjbar et al., 2005). In addition, numerous authors have observed that pyrethroid exposure impairs enzymatic antioxidants (Jebur et al., 2014; Syed et al., 2016), implying that the antioxidant defense system is unable to cope with the flood of ROS created by β-Cyf exposure. This occurs as a result of enzyme depletion or inhibition during the breakdown of free radicals, or as a result of direct inhibition of enzymes by β-Cyf.

The observed changes in hepatic enzymes activity (ALT, AST, ALP, and LDH) in β-Cyf treated rats could be attributable to LPO, which disrupts the integrity of cellular membranes, allowing cytoplasmic enzymes to leak into the circulation following hepatocellular injury (Gokcimen et al., 2007). In parallel, several authors concluded that pyrethroids exposure caused changes in enzymes activity since they have the potency to induce hepatic injury and physiological impairments (El-Demerdash, 2007; Jebur et al., 2014; Verma et al., 2016). Protein is also one of the most sensitive cell components to free radical damage. Excessive protein loss via nephrosis, or induction of proteolytic enzymes activity or degradation, were the main causes of the observed drop in protein content (Chatterjea and Shinde, 2002). In addition, a change in normal liver function could be linked to the significant drop in total protein content and albumin in β-Cyf treated rats (Bhushan et al., 2013). Furthermore, an increase in total bilirubin may be caused by decreased liver output or occlusion of the bile ducts as a result of liver cell failure (Sanjiv, 2002).

In the current study, TNF-α and IL-6 gene expressions were significantly elevated in the β-Cyf treated rats. Similarly, increased expression of the interleukin-6 receptor (IL-6R) and tumor necrosis factor receptor superfamily (TNFRSF10A) genes, as well as inflammation, have been related to β-Cyf (Abdou et al., 2017). Furthermore, pyrethroid-produced free radicals are thought to play a role in the generation of proinflammatory cytokines like IL-12, INF-γ, and TNF-α (Hussein and Ahmed, 2016). ROS are also potent activators of the NF-ĸB transcription factor, which is involved in innate immunity and inflammation. TNF-α, IL-6, and other inflammation-related genes are all upregulated when NF-ĸB is activated (Tabrez et al., 2014). Furthermore, previous studies showed that the increase of IL-6 and TNF-α, associated with up-regulation of NF-kappaβ, IL-1β, IL-10, P53, and pro-apoptotic gene Bax (Ahmed et al., 2017; Nna et al., 2018). Hepatic protein depletion elucidates disruption to membrane integrity, which in turn indicates the admission of a toxicant into the cell. Both the toxicant and its metabolic intermediates can cause a range of problems within the cell, including the release of numerous hydrolytic enzymes such as nucleases and proteases making cells more susceptible to chromosomal damage and cell cycle disruption (Singh and Saxena, 2002). Also, the decline in hepatic protein may be affecting genetics by modifying the cell cycle, resulting in hepatic cell proliferation (Omotuyi et al., 2006). Furthermore, various authors have documented that pesticides can damage DNA in hepatocytes, lymphocytes, and other cells in the body, which is consistent with the increase in tail length and DNA percent of rat liver intoxicated with β-Cyf (Cortes-Gutierrez et al., 2011; Hussain et al., 2011). The harmful influence of β-Cyf on rat liver DNA is due to its buildup in liver tissue, which leads to an increase in free radical generation, oxidative stress, and apoptosis (Hussein and Ahmed, 2016).

Oral BEO administration was able to reduce LPO and increase antioxidant status because of its wide range of phenolic components and natural products (Kwon et al., 2019). The increase in radical scavenging activity of BEO in this study was most likely due to its significant component estragole. The most prominent component in Egyptian basil leaves essential oil, according to Chenni et al. (2016), is linalool, and the chemicals observed in this study were equivalent to those found by Falowo et al. (2019). The differences in total phenolic compounds (TPC) between our study and others can be attributed to the different environmental circumstances (Ahmed et al., 2019). According to the findings, BEO has a high reducing scavenging activity and a high DPPH radical scavenging ability, indicating that the oxygenated phenolic monoterpene and a mixture of mono and sesquiterpene hydrocarbons are the most likely culprits (Pripdeevech et al., 2010). According to Ofem et al. (2012) and Zangeneh et al. (2019), BEO improved hematological parameter disorders, and this effect may be attributed to the proportion of iron present in basil (Nworgu et al., 2013), which can stimulate the production of RBC and promote blood Hb level to recover their deficiency induced by β-Cyf.

LPO, alterations in enzymatic and non-enzymatic antioxidants, and liver biomarkers induced by β-Cyf were all mitigated by BEO supplementation. This is due to basil’s high concentration of phenolic components as phenolic acids and flavonoids, which have redox characteristics and can scavenge free radicals (Ahmed et al., 2019). Basil is beneficial in the treatment of hepatic fibrosis, with a significant decrease in hydroxyproline deposition in hepatocytes confirming the alleviation of fibrotic changes in the liver and promoting liver regeneration in fibrotic rats (Parola and Robino, 2001). Basil’s antifibrotic properties are related to its flavonoids which have high antioxidant capacity. In addition, Basil extract decreased the gene expression of fibrosis-related extracellular matrix components and hindered the conversion of hepatic stellate cells to myofibroblasts (Salmah et al., 2005). Also, oral administration of BEO basil extracts reduced inflammation in rats exposed to β-Cyf. These findings are in line with several findings that reported that basil possesses anti-inflammatory properties via blocking inflammatory mediator receptors and cell migration to stimulation sites (Aye et al., 2019; Takeuchi et al., 2020).

In general, according to our findings, β-Cyf exposure is linked to increased oxidative stress and the production of reactive oxygen species (ROS), which interact with macromolecules in liver tissue and cause lipid peroxidation, protein oxidation, fibrosis, hematotoxicity, depletion in enzymatic and non-enzymatic antioxidants, increased pro-inflammatory cytokines, DNA damage, and arrested cells in the G0/G1 phase. So, the administration of basil leaves essential oil as a phytochemical drug, has been documented to have a plethora of phenolic constituents that may explain its strong free radical scavenging effect leading to the modulation of the observed β-Cyf toxicity verifying our hypothesis which is BEO could ameliorate the toxic effect of β-Cyf due to its high antiradical and antioxidant activity.

## Conclusion

β-cyfluthrin induced oxidative stress in rat liver tissue, leading to disturbances in antioxidant status and liver function biomarkers. Pre-treatment with BEO improved most of the enzymatic activities lost due to β-Cyf toxicity. BEO displayed great DPPH radical scavenging capacity and powerful antioxidant activity because of its phenolic components that act as strong free radicals’ scavengers. Furthermore, BEO exhibits anti-inflammatory and antioxidant capabilities that can alleviate β-Cyf-induced hepatotoxicity in rats by inhibiting oxidative stress, DNA damage, inflammation, and cell cycle arrest. So, basil is a low-cost botanical drug that is widely available and has been shown to have a high antioxidant capacity.

## Data Availability

The original contributions presented in the study are included in the article/Supplementary Material, further inquiries can be directed to the corresponding author.
